# The Impact of Cefepime and Ampicillin/Sulbactam on Preventing Post-Cesarean Surgical Site Infections, Randomized Controlled Trail

**DOI:** 10.3390/antibiotics12121666

**Published:** 2023-11-27

**Authors:** Mona A. Abdelrahman, Asmaa Zaki, Sara A. M. Salem, Heba F. Salem, Ahmed R. N. Ibrahim, Ahmed Hassan, Marwa O. Elgendy

**Affiliations:** 1 Department of Clinical Pharmacy, Faculty of Pharmacy, Beni-Suef University, Beni-Suef 62511, Egypt; 2 Department of Clinical Pharmacy, Faculty of Pharmacy, Nahda University (NUB), Beni-Suef 62764, Egypt; asmaa.zaki@nub.edu.eg (A.Z.); marwa.elgendy@nub.edu.eg (M.O.E.); 3 Department of Obstetrics and Gynecology, Faculty of Medicine, Beni-Suef University, Beni-Suef 62521, Egypt; sara_abdallah100@yahoo.com; 4 Department of Pharmaceutics & Industrial Pharmacy, Faculty of Pharmacy, Beni-Suef University, Beni-Suef 62511, Egypt; heba_salem2004@yahoo.co.uk; 5 Department of Pharmaceutics and Industrial Pharmacy, 6 October Technological University, Giza 12585, Egypt; 6 Department of Clinical Pharmacy, College of Pharmacy, King Khalid University, Abha 61421, Saudi Arabia; aribrahim@kku.edu.sa; 7 Department of Clinical Pharmacy, Faculty of Pharmacy, University of Sadat City, Sadat City 32897, Egypt; ahmhassancp@gmail.com; 8 Department of Clinical Pharmacy, Beni-Suef University Hospitals, Faculty of Medicine, Beni-Suef University, Beni-Suef 62521, Egypt

**Keywords:** surgical site infection, cesarean, cefepime, Unictam

## Abstract

Over the previous three decades, the rate of caesarean sections performed worldwide has grown exponentially. In comparison to a vaginal birth, the risk of all postpartum infections is higher with a cesarean section. One of the key factors contributing to maternal morbidity is the development of infectious complications in the surgical site after a caesarean section. The primary goal of the research was to compare the efficiency of using ampicillin/sulbactam (AMS) and cefepime (CEF) to reduce the incidence of surgical site infections (SSI) following caesarean delivery. This prospective randomized study was conducted among 200 pregnant women scheduled for elective cesarean section. They were collected from the Obstetrics and Gynecology department of Beni-Suef University Hospital, and then they were randomly assigned into two groups. Group (A) received cefepime 30 min before and 12 h after cesarean delivery, while group (B) received ampicillin/sulbactam 30 min before and 12 h after cesarean delivery. The groups were matched regarding the baseline women characteristics. Comparing the cefepime to the ampicillin/sulbactam revealed that the cefepime significantly decreased superficial SSI from 27% to 14% (0.023). A significant decrease was observed in deep SSI with cefepime compared to ampicillin/sulbactam from 24% to 13% (*p*-value 0.045). Interestingly, when the cefepime was compared to the ampicillin/sulbactam, we noted that the incidence of endometritis significantly decreased from 13% to 5% (*p* = 0.048). A noted decrease in post-operative fever in cefepime as compared to ampicillin/sulbactam from 18% to 13% (*p*-value = 0.329). Receiving prophylactic cefepime pre- and post-cesarean delivery significantly decreases post-operative wound infection and endometritis.

## 1. Introduction

A cesarean section (CS) can be a life-saving surgical procedure to deliver a healthy baby to a healthy and satisfied mother. Globally, the CS rate has increased, and it is estimated to account for about 19–20% of all births [1]. Infectious morbidities are 5–20 times more common following CS than vaginal delivery [2]. The burden of post-CS infection is greater in developing countries than in developed countries [3]. In developed countries, surgical site infection (SSI) complicates about 3.7–9.9% of CS births, while it ranges between 7.1% and 19% in Africa [3].

Infections at the surgical site (SSIs) are major causes of morbidity and mortality for patients undergoing any kind of surgery. Morbidity and mortality rates rise along with the length of hospital stay, medical expenses, and other factors related to these infections. Compared to vaginal births, maternal infectious morbidity increases to eight times after caesarean delivery [4].

Despite the widespread use of prophylactic antibiotics, post-operative infectious morbidity still complicates cesarean deliveries. Our challenge is to minimize the occurrence of this common cause of post-cesarean delivery morbidity (endometritis and wound infections). Peri-operative antimicrobial prophylaxis has been demonstrated in numerous studies to be beneficial in preventing post-surgical infection following caesarean delivery. Some authors argue that the most effective regimen has not been established yet [5]. For example, cephalosporins have been widely used for antimicrobial prophylaxis during cesarean delivery [6]. However, in one study it was shown that a broader combination of cefazolin and azithromycin provided better efficacy with regard to post-operative infectious disease morbidity and duration of hospitalization when compared to cefazolin alone [7]. In a recent randomized trial, it was shown that ampicillin/sulbactam and first- and second-generation cephalosporins like cefoxitin, cefazoline, and cefuroxime have similar efficacy on post-operative cesarean section infections [6,8,9,10]. Moreover, in an obstetrical study, it fared better than ampicillin alone in preventing post-cesarean infection in women who had ruptured membranes [11]. It is not clear whether any one particular antibiotic is superior [12,13]. To the best of our knowledge, no direct comparison between ampicillin/sulbactam AMS and cefepime CEF has been performed to date regarding their use as prophylaxis in cesarean delivery; therefore, this study aimed to evaluate the efficacy of employing cefepime (CEF) versus ampicillin/sulbactam (AMS) in lowering the rate of post-cesarean surgical site infections. 

## 2. Results

### 2.1. Demographic and Pregnancy-Related Data

The studied subjects were bioequivalent comparable as regarding the baseline maternal characteristics (*p* > 0.05) as presented in Table 1. Statistical analysis of data noted a non-significant difference between both groups regarding age, weight, pre-operative haemoglobin, gestational age, and cesarean section duration (*p* > 0.05).

### 2.2. Comparative Results Regarding the Occurrence of Infections

**Figure 1:**
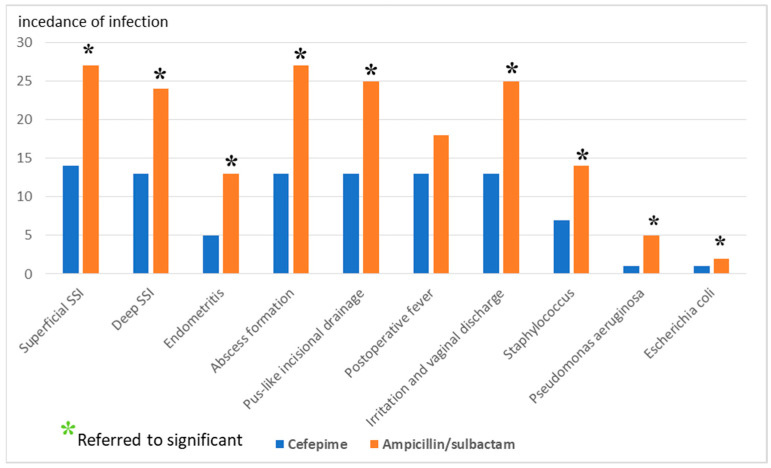
Comparison between the CEF and AMS regarding the post-operative infection. * *p*-value is significant.

The results are presented in Table 2 and graphically illustrated in Figure 1. 

Regarding the incidence of surgical site infection (SSI), the CEF significantly decreases superficial SSI (from 27% to 14%, *p*-value 0.023) compared to the AMS. Moreover, a significant decrease in deep SSI upon receiving the CEF compared to the AMS (from 24% to 13%, *p*-value 0.045). Receiving the CEF showed a significant decrease in irritation and vaginal discharge by 12% compared to the AMS (*p*-value 0.031). Interestingly, when compared to the AMS, women who received the CEF had a significantly lower incidence of endometritis (*p* = 0.048). Women receiving the CEF showed a significant reduction in abscess formation (from 27% to 13%, *p*-value 0.013) compared to the AMS. On the other hand, neither receiving the CEF nor AMS resulted in a statistically significant difference in post-operative fever (*p* = 0.329).

Shifting to Table 3, the infection rate among the study participants was a high burden among the AMS group when compared to the CEF group; *Staphylococcus aureus* was the most common microbial isolate (70%). *Staphylococcus aureus* was isolated more in the AMS group (66.66%) than in the CEF arm (33.33%). The second most common bacterial isolate was the *Pseudomonas aeruginosa*, which was isolated in 20% of women (16.66% in the CEF group vs. 83.33% in the AMS group). *Escherichia coli* was 10% (33.33% vs. 66.66%).

Table 4 shows that after adjustment for age, gestational age, pre-operative haemoglobin, and duration of operation in the presence of CEF use (referenced by AMS), it was demonstrated that the use of CEF significantly decreased the post-operative infection probability with OR (95%CI) equals 0.400 (0.185, 0.865).

This figure shows that there was a significant difference between overall post-operative infections in the CEF vs. AMS group.

## 3. Discussion

Infectious morbidity after cesarean delivery can have a tremendous impact on the postpartum woman’s return to normal function and her ability to care for her baby [14,15]. This randomized trial was mainly designated to compare the efficacy of using prophylaxis ampicillin/sulbactam (AMS) or cefepime (CEF) undergoing cesarean delivery. Surgical site infection (SSI) was investigated as the primary outcome; therefore, it was determined in the form of superficial and deep SSI, endometritis, abscess formation, pus-like incisional drainage, irritation and vaginal discharge, and post-operative fever [16,17].

In this study the incidences of superficial and deep SSI in the AMS group were more significant than in the CEF group, (27% and 24% vs. 14% and 13%, respectively *p*-value < 0.05). This result was higher than that conducted by Joel M. Marwa1 et al. who found superficial SSI was 4.27% in CEF among 117 participants [18]. However, in the study of Victor N. Mivumbi et al., the SSI that occurred with ampicillin/sulbactam was 4.6% [19]. Also, in another study conducted by Eleftherios Ziogos1et al, the incidence of post-operative infections developed in 8.8% of women who received AMS [20]. 

To the best of our knowledge, no direct comparison between AMS verse CEF has been performed to date regarding their use as prophylaxis in cesarean delivery. In this study, CEF showed a better effect as a protective agent against surgical site infections than AMS. However, a study conducted by Eleftherios Ziogos et al. found there is no superiority for the AMS regimen over cefuroxime in decreasing post-cesarean infectious morbidity [20]. In addition, Peechakara BV et al., reported that AMS has an efficacy similar to third-generation cephalosporins in treating skin infections [21].

This may be attributed to CEF a fourth-generation cephalosporin with broad-spectrum efficacy against both Gram-negative and Gram-positive bacteria, which exhibits a decreased bacterial resistance relationship in contrast to those second-generation and third-generation cephalosporin [22]. Moreover, CEF has an additional quaternary ammonium group, allowing them to better penetrate the outer membrane of gram-negative bacteria. CEF can cover *Streptococcus pneumoniae* and methicillin-sensitive *Staphylococcus aureus* (MSSA) very importantly, can cover *Pseudomonas aeruginosa* [23].

Endometritis was reported in 40–70% of patients before prophylactic antibiotic therapy, the use of prophylactic antibiotics reduced the incidence of endometritis by 50% [24,25].

Comparing the incidence of endometritis CEF vs. AMS revealed that CEF significantly decreased endometritis (from 13% to 5% *p*-value 0.048). These results are slightly higher than those found in the meta-analysis which indicated that the incidence of postpartum endometritis was 7.4% following the AMS regimen [24]. A study conducted by Eleftherios Ziogos et al reported the incidence of post-operative endometritis developed was 6.6% of patients who received AMS [20].

The present data demonstrated that the most predominant bacteria causing surgical site infections was *Staphylococcus aureus* was the most common microbial isolate (70%) and the second most common bacterial isolate was the *Pseudomonas aeruginosa*, which was isolated in 20% of women. These findings are in close agreement with that presented by Daniela Călina et al., who observed that the most prevalent bacterial species isolated from wound infections in patients undergoing surgery caused by *S. aureus* 50.72% and *Pseudomonas aeruginosa* 10.05% [26]. In contrast to a study conducted by Bazira Joel et al. who found *Klebsiella pneumoniae* caused 50% of the SSIs followed by *Staphylococcus aureus* at 27.8% and *E. coli* [27]. This difference in the distribution of bacterial species might be due to variation in common hospital-acquired pathogens, and infection prevention and control policies and guidelines across countries [28,29,30]. 

Receiving CEF showed a significant decrease in *Pseudomonas aeruginosa* by 66.67% from 83.33 to 16.66% as compared to AMS. 

It must be taken into account that Gram-negative bacteria are more resistant than Gram-positive bacteria, due to their distinctive structure, and cause significant morbidity and mortality worldwide [31]. CEF has an additional quaternary ammonium group, allowing it to better penetrate the outer membrane of gram-negative bacteria [23]. Furthermore, the fourth generation of cephalosporins like CEF, is the most effective β-lactams used in the treatment of *P. aeruginosa*. Resistance to these antibiotics is mediated by β-lactamases which destroy the amide bond of the β-lactam ring and make the antibiotics ineffective [31].

Considering this study, CEF showed a better effect as a protective agent against surgical site infections than AMS. These findings are supported by The National Institute for Clinical Excellence (NICE) and the World Health Organization (WHO) which recommend that a prophylactic, broad-spectrum cephalosporin is given before skin incision as this reduces the risk of SSIs by 50% [22].

It is important to take into account that the choice of an antibiotic should be based on its antibacterial spectrum and the indication [32,33]. As mentioned previously the most common microorganisms causing SSI reported in this study were *Staphylococcus aureus* and *pseudomonas aeruginosa.* It is worth noting that *Staphylococcus aureus* is a gram-positive bacteria [34,35,36]. *Pseudomonas aeruginosa*, belonging to the Pseudomonadaceae family, is a Gram-negative, rod-shaped, motile, aerobic bacteria [37].

Considering this study, CEF showed a better effect as a protective agent against surgical site infections than AMS. This could be attributed to the presence of the bacterial enzymes which inactivate penicillin in AMS (that is also known as penicillinase-resistant penicillin) [38,39]. This resistance may have developed due to the empirical use of AMS in hospitals. These findings are in close agreement with prior studies which showed that CEF is more effective than other broad-spectrum-lactams and non-lactams [22,40].

The lower efficacy of AMS than CEF could be explained by the lower efficacy of AMS against gram-negative bacteria than gram-positive bacteria. Some antibiotics, such as β-lactam antibiotics that target peptidoglycan in the bacterial cell wall, are ineffective against gram-negative bacteria because their chemical properties prevent them from using these pathways to effectively penetrate the outer membrane [41,42,43].

This study also has some limitations. First, we conducted the trial at a single site, which raises a question about the potential generalizability of our findings to other hospital settings. Furthermore, the study was in a single centre and for this combination only a few eligible isolates were assessed. Second, it is not representative as it includes a small number of isolates. Hence, further studies should be performed on a large scale with a larger number of patients and longer treatment durations to confirm the certainty of these results.

## 4. Materials and Methods

### 4.1. Study Design

**Figure 2:**
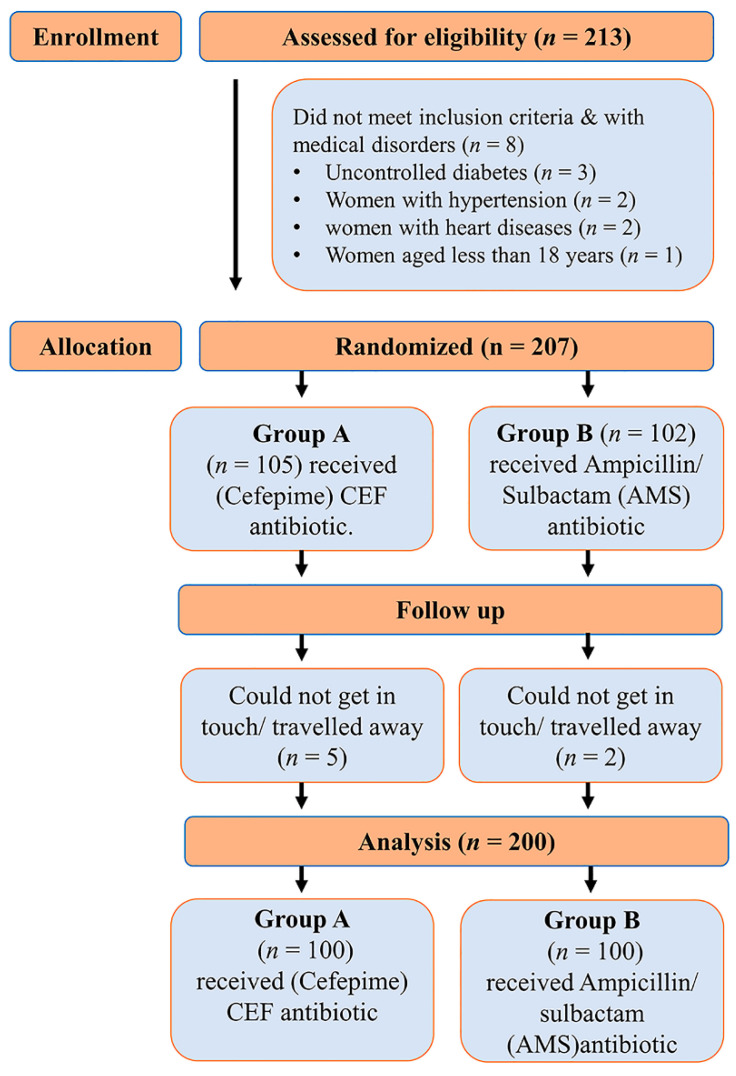
Flow chart of participants’ enrollment process of study.

A prospective randomized clinical study was conducted in the department of Obstetrics and Gynaecology of Beni-Suef University Hospital from September 2020 to February 2022. A total of 213 women were assessed for eligibility, 13 of which were excluded as illustrated in Figure 2. A total of 200 pregnant women were eligible for elective cesarean sections (CS). The overall median duration of post-operative follow-up was from 10 days to one month to determine the surgical site infection (SSI). All the participants fulfilled the inclusion criteria.

The patients were divided into two groups. Group (A): Pregnant women (*n* = 100) received IV cefepime (CEF) (Maxipime^®^1000 mg PHARCO Pharmaceuticals, Alexandria Egypt) 30 min before cesarean delivery (CD) and 12 h after CD, and Group (B): Pregnant women (*n* = 100) received IV ampicillin/sulbactam (AMS) (Unictam^®^1500 mg Medical Union Pharmaceuticals MUP, Obour City, Egypt) 30 min before CD and 12 h after CD.

### 4.2. Sample Size

The power of the sample size was estimated using power software version 3.1.9.2 (post hoc detection of power) based on the most important parameters post-cesarean infection, it was 95% depending on effect size, α = 0.05, normality of data and 2-tailed assays.

### 4.3. Study Population

#### 4.3.1. Inclusion Criteria

Pregnant women within the age group of more than 18 years.

Pregnant women with elective cesarean delivery.

Previous and primary cesarean delivery.

#### 4.3.2. Exclusion Criteria

Severe hepatic disease women.

Women aged less than 18 years.

Emergent cesarean deliveries.

Women with medical disorders such as pre-gestational diabetes, anaemia, hypertension, or preeclampsia.

Women who had a skin infection adjacent to the operative site.

### 4.4. Study Intervention

Women undergoing elective CS were administered prophylactic antibiotics, either the CEF for group (A) or the AMS for group (B), 30 min before and 12 h after CS. According to hospital protocol, the circulating nurse cleaned and prepped the skin by following the manufacturer’s guidelines. The antiseptic (Povidone-Iodine) was opened, and the surgical site was scrubbed with it. A period of waiting for 3 min was allowed between the application of the antiseptic agent and skin incision to allow the adhesive surgical drapes to stick properly over dry skin.

The skin of all eligible individuals was aseptically treated in the same technique, using the same materials in an equal amount to control variances. All the enrolled participants received the same health education and wound care advice from the professional health team. 

### 4.5. Data Collection

For all cases, data were collected the following:

I. Structured interview

Part A: Participants demographic and obstetric data

To determine (name, age, weight, gestational age per week, type of cesarean delivery (1 ry—the first cesarean in mother’s life, repeat) and post-operative hospital stay)

Part B: Participants clinical profile

To assess (pregnancy infection, pre-gestational diabetes, gestational diabetes, hypertension, preeclampsia, pre-operative haemoglobin)

II. Postpartum follow-up

Hospital policy is to discharge the women on the second post-operative day after C-section if the women are in good post-operative condition. Women with surgical site infection (SSI) had a lengthier hospital stay but all did well during follow-up. Participants return on the 10th day for reassessment/evaluation.

The follow-up was conducted in the outpatient department during hospital visits (on the 10th postpartum day). SSI was diagnosed by a gynaecologist or attending physician. SSI was determined based on fulfilling one of the criteria adapted from the Centers for Disease Control and Prevention (CDC) [12]. 

Culture-based microbiological methods were used to identify causal agents in post-operative wounds. Material was taken using sterile swabs from infected women with SSI and then forwarded to the Department of Microbiology. 

Patients with SSI were advised to use sterile saline for wound cleansing up to 48 h after surgery. Additionally, patients were told that they may shower safely 48 h after surgery. Patients were also advised to use tap water for wound cleansing after 48 h if the surgical wound has separated or has been surgically opened to drain pus. They were instructed not to use Eusol and gauze, moist cotton gauze, or mercuric antiseptic solutions to manage surgical wounds that are healing by secondary intention.

The second follow-up was done by phone calls. The telephone calls were made to women who were unable to attend the outpatient clinic between 10 days and one month postpartum. The phone conversation asked about the following: (1) Were you diagnosed with SSI within 10 days of your cesarean section?; and (2) Were you admitted to the hospital again? If so, why were you readmitted? 

Moreover, a checklist was performed to record the postpartum follow-up (Table 5) (from 10 to one month postpartum). It was designed to assess the presence or absence of the post-CS surgical site of infection and endometritis after cesarean delivery). The clinical features of the wound such as pain, redness, swelling, warm skin around the wound, yellow or green discharge, and fever were considered for clinical diagnosis of surgical site infection. Then, comparing different responses between the groups was accomplished. 

### 4.6. Outcome Measures

The surgical site infection is the outcome measure in this study and was detected in the form of superficial SSI, deep SSI, endometritis, abscess formation, pus-like incisional drainage, irritation and vaginal discharge, post-operative fever, and positive blood culture.

The patients were followed up daily for SSI for two weeks or until complete recovery to detect the rate of SSI incidence.

## 5. Conclusions

Receiving the prophylactic cefepime pre- and post-cesarean delivery significantly decreases post-operative wound infection and endometritis.

## Figures and Tables

**Table 1:** Baseline characteristics of the cefepime (CEF) vs. ampicillin/sulbactam (AMS) group.

Items	CEF Group (no = 100) Mean ± SD	AMS Group (no = 100) Mean ± SD	*p*-Value
Age (year)	29.3 ± 4.6	28.1 ± 5.6	0.109
Weight (kg)	76.5 ± 12.8	74.7 ± 14.1	0.344
Gestational age (week)	39.7 ± 1.8	39.1 ± 2.5	0.053
Pre-operative haemoglobin (g/dl)	11.4 ± 1	11.2 ± 0.9	0.075
Cesarean section duration (minutes)	40.7 ± 7.2	41.6 ± 4.7	0.296

**Table 2:** Comparison between the cefepime and ampicillin/sulbactam groups regarding the occurrence of infections.

Items	CEF Group (no = 100)	AMS Group (no = 100)	*p*-Value
Superficial SSI			
No	86 (86.0%)	73 (73.0%)	0.023 *
Yes	14 (14.0%)	27 (27.0%)	
Deep SSI			
No	87 (87.0%)	76 (76.0%)	0.045 *
Yes	13 (13.0%)	24 (24.0%)	
Endometritis			
No	95 (95.0%)	87 (87.0%)	0.048 *
Yes	5 (5.0%)	13 (13.0%)	
Abscess formation			
No	87 (87.0%)	73 (73.0%)	0.013 *
Yes	13 (13.0%)	27 (27.0%)	
Pus-like incisional drainage			
No	87 (87.0%)	75 (75.0%)	0.031 *
Yes	13 (13.0%)	25 (25.0%)	
Post-operative fever			
No	87 (87.0%)	82 (82.0%)	0.329
Yes	13 (13.0%)	18 (18.0%)	
Irritation and vaginal discharge			
No	87 (87.0%)	75 (75.0%)	0.031 *
Yes	13 (13.0%)	25 (25.0%)	
Culture (SSI)			
Negative	91 (91.0%)	79 (79.0%)	<0.001 *
Positive	9 (9.0%)	21 (21%)	
*Staphylococcus*	7/9 (77%)	14/21 (66.66%)	0.001 *
*Pseudomonas aeruginosa*	1/9 (11.11%)	5/21 (23.8%)	<0.001 *
*Escherichia coli*	1/9 (11.11%)	2/21 (9.52%)	<0.001*

* *p*-value is significant.

**Table 3:** Microbial isolation of the cefepime group vs. ampicillin/sulbactam group.

Items	Total Isolated Organism	CEF Group	AMS Group
*Staphylococcus*	21/30 (70%)	7/21 (33.33%)	14/21 (66.66%)
*Pseudomonas aeruginosa*	6/30 (20%)	1/6 (16.66%)	5/6 (83.33%)
*Escherichia coli*	3/30 (10%)	1/3 (33.33%)	2/3 (66.66%)

**Table 4:** Multivariable binary logistic regression analysis for risk factors affecting post-operative infection in CEF or AMS use.

Independent Variables	*p*-Value	OR	95% CI. for OR	
			Lower	Upper
CEF use	0.020 *	0.400	0.185	0.865
Age (years)	0.805	1.009	0.940	1.082
Gestational age (weeks)	0.325	1.285	0.780	2.119
Pre-operative haemoglobin (gm/dl)	0.991	0.998	0.690	1.444
Cesarean duration (minutes)	0.732	0.989	0.929	1.053

* *p*-value is significant OR = Odds ratio CI = confidence interval.

**Table 5:** A checklist to follow-up postpartum SSI [13].

NO.	Questions
1.	Was the redness spreading away from the wound? (erythema/cellulitis)
2.	Was the area nearby the wound warmer than the close skin?
3.	Was any part of the wound with thick and yellow/green fluid (pus/purulent exudate)?
4.	Has the wound been painful to touch?
5.	Have you had, or felt like you have had, a raised temperature or fever? (fever > 38 °C)
6.	Has anything been put on the skin to cover the wound? (dressing)
7.	Have you been return to the hospital for treatment of any problem with the wound?
8.	Have you been given antibiotics for a problem with your wound?
9.	Has your wound been drained? (drainage of abscess)

## Data Availability

Data is contained within the article.
